# Significant *In Vivo* Anti-Inflammatory Activity of Pytren4Q-Mn a Superoxide Dismutase 2 (SOD2) Mimetic Scorpiand-Like Mn (II) Complex

**DOI:** 10.1371/journal.pone.0119102

**Published:** 2015-03-05

**Authors:** Carolina Serena, Enrique Calvo, Mari Paz Clares, María Luisa Diaz, Javier U. Chicote, Raúl Beltrán-Debon, Ramón Fontova, Alejandro Rodriguez, Enrique García-España, Antonio García-España

**Affiliations:** 1 Research Unit, Hospital Joan XXIII, Institut d’Investigació Sanitaria Pere Virgili (IISPV), Universitat Rovira i Virgili, Tarragona, Spain; 2 Instituto de Ciencia Molecular, Departamento de Química Inorgánica, Universidad de Valencia, Valencia, Spain; 3 Pathology Department, Hospital Joan XXIII, Institut d’Investigació Sanitaria Pere Virgili (IISPV), Universitat Rovira i Virgili, Tarragona, Spain; 4 Rheumatology Department, Hospital Joan XXIII, Institut d’Investigació Sanitaria Pere Virgili (IISPV), Universitat Rovira i Virgili, Tarragona, Spain; 5 Critical Care Department, Hospital Joan XXIII, Institut d’Investigació Sanitaria Pere Virgili (IISPV), Universitat Rovira i Virgili, Tarragona, Spain; 6 CIBER de enfermedades respiratorias (CIBERES), ISC III, Bunyola, Palma de Mallorca, Spain; University of Valencia, SPAIN

## Abstract

**Background:**

The clinical use of purified SOD enzymes has strong limitations due to their large molecular size, high production cost and immunogenicity. These limitations could be compensated by using instead synthetic SOD mimetic compounds of low molecular weight.

**Background/Methodology:**

We have recently reported that two SOD mimetic compounds, the Mn^II^ complexes of the polyamines Pytren2Q and Pytren4Q, displayed high antioxidant activity in bacteria and yeast. Since frequently molecules with antioxidant properties or free-radical scavengers also have anti-inflammatory properties we have assessed the anti-inflammatory potential of Pytren2Q and Pytren4Q Mn^II^ complexes, in cultured macrophages and in a murine model of inflammation, by measuring the degree of protection they could provide against the cellular injury produced by lipopolisacharide, a bacterial endotoxin.

**Principal Findings:**

In this report we show that the Mn^II^ complex of Pytren4Q but not that of Pytren2Q effectively protected human cultured THP-1 macrophages and whole mice from the inflammatory effects produced by LPS. These results obtained with two molecules that are isomers highlight the importance of gathering experimental data from animal models of disease in assessing the potential of candidate molecules.

**Conclusion/Significance:**

The effective anti-inflammatory activity of the Mn^II^ complex of Pytren4Q in addition to its low toxicity, water solubility and ease of production would suggest it is worth taking into consideration for future pharmacological studies.

## Introduction

The normal cellular metabolism of aerobic organisms generates, as a by-product, deleterious reactive oxygen species (ROS) including, among others, superoxide radicals that need to be eliminated. Superoxide radicals (O_2_^-^) are deactivated in an initial step by superoxide dismutase (SOD) enzymes [1]. The insufficient deactivation of superoxide radicals has been implicated in the pathogenesis of several acute and chronic diseases with an inflammatory component such as, atherosclerosis, obesity, diabetes, cancer and sepsis [2–5]. Purified SOD enzymes of bovine and human origin had shown promising pharmacological activity, first in the treatment of some of these disorders such as rheumatoid arthritis and osteoarthritis, chronic obstructive pulmonary diseases, urinary tract inflammatory disease, and second in providing protection against the side effects of chemotherapy and radiation therapy [2, 6, 7]. However, purified enzymes have several limitations due to their high molecular size, production cost and antigenic activity in the case of non-human proteins [6, 8, 9]. In this regard, synthetic SOD mimetic compounds of low molecular weight could compensate the limitations of purified enzymes due to their lack of antigenicity, tissue penetrance, higher stability in solution, longer half-life, and lower production cost [6, 8].

**Fig 1:**
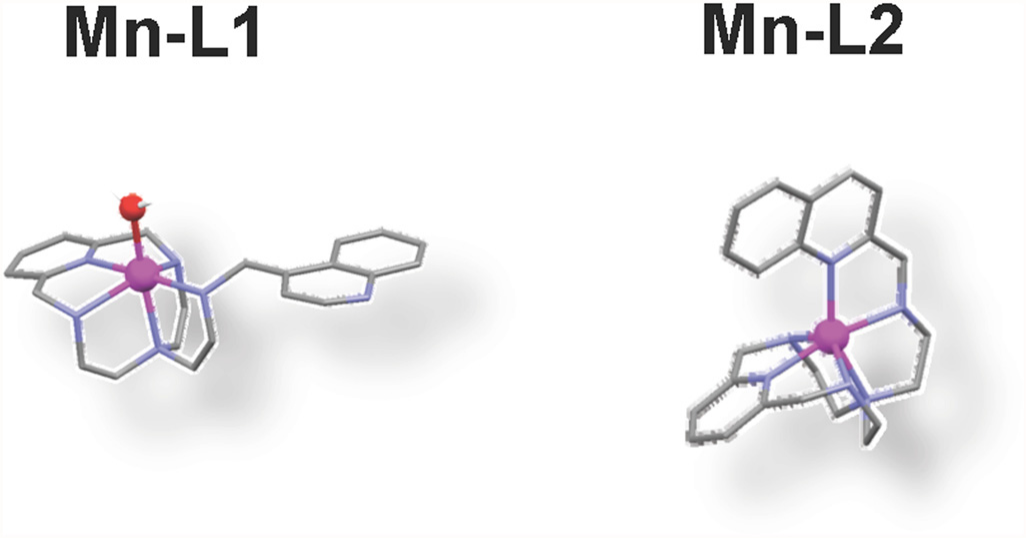
Drawing of Pytren4Q-Mn (Mn-L1) and Pytren2Q-Mn (Mn-L2). Stick and ball structure of Mn-L1 and Mn-L2. In red oxygen, pink manganese and blue nitrogen. Notice that Mn-L1 has a water molecular in its active center which plays an important role in regulating the redox potential and in assisting the proton transfer processes occurring in superoxide anion dismutation [11].

The specific removal of superoxide anions can modulate the course of inflammatory processes as described using SOD enzymes in pharmacology [6]. We decided to assess the anti-inflammatory potential of two new, water-soluble, non-steroid, SOD mimetics the Mn^II^ complexes of of Pytren4Q (Mn-L1) and Pytren2Q (Mn-L2) (Fig. 1) which have shown high anti-oxidant activity in SOD-deficient bacteria and yeast [10, 11,12]. For this purpose, we used cellular and animal models of inflammation. In these models the anti-inflammatory potential of a molecule can be assessed as the degree of protection it provides against the cellular injury produced by lipopolisacharide (LPS), a gram-negative bacterial endotoxin, that induces the expression of pro-inflammatory mediators [13, 14].

In this report we show that Mn-L1 effectively protected human cultured THP-1 macrophages and whole mice from the inflammatory effects produced by LPS. Mn-L1 consistent anti-inflammatory activity, low toxicity, water solubility and stability suggest Mn-L1 could have real therapeutic potential and makes Mn-L1 worth taking into consideration for future pharmacological studies.

## Materials and Methods

### Reagents

Metal ligands Pytren4Q (L1) and Pytren2Q (L2) were synthesized as reported previously [10]. Phorbol 12-myristate 13-acetate (PMA), *LPS (from Escherichia coli 0111*: *B4*), L-Ascorbic (L-ASC), and Resveratrol (RSV) were supplied by Sigma-Aldrich (St. Louis, MO, USA). Antibodies were obtained from the following vendors: akt, phospho-Akt (Ser473), ERK1/2, phospho-ERK1/2 (Thr202/Tyr204), p38 MAPK, phospho-p38 MAPK (Thr180/Tyr182), JNK, phospho-JNK (Thr183/Tyr185), IKK and phospho-IKK (Ser176) (Cell Signaling Technology, Inc. Beverly, MA, USA), ß-actin AC-15 (Sigma-Aldrich; St. Louis, MO, USA), Anti-F4/80 MCA497RT, Serotec (Oxford, UK).

### Cell culture

THP-1 human monocytes cells (ATCC, Rockville, USA) were cultured at 37°C in 95% humidified air/ 5% CO_2_ in RPMI-1640 medium containing 10% (v/v) fetal bovine serum (FBS), supplemented with 2 mM L-Glutamine, 100 U/ml penicillin, and 100 μg/ml streptomycin. THP-1 monocytes were seeded at 9 x 10^5^ cells/well in 6-well-plates and differentiated to macrophages with 200 nmol/L of PMA for 3 days plus a further 2 days’ incubation in fresh medium without PMA prior to starting the treatment as described in the figure legends [15].

Monkey kidney VERO cells (ATCC, Rockville, USA) were cultured in Dulbecco’s Modified Eagle Medium (DMEM) supplemented with 2 mM L-glutamine and 10% FBS, 100 U/ml penicillin and 100 μg/ml streptomycin.

### RNA preparation and reverse transcriptase polymerase chain reaction (RT-PCR)

Total cellular RNA was isolated using an RNeasy Mini Kit, from QIAGEN. From each sample, 500 ng of RNA was reverse-transcribed using the First Strand cDNA Synthesis Kit for RT-PCR (AMV) from Roche Applied Science. PCR analyses were performed on aliquots of the cDNA preparations to detect expression of *TNF-α*, *IL-6*,*IL-8*, *IL-1ß* and *PTGS2*, and *PPIA*, *GADPH*, *RLPO* as housekeeping genes using a thermal cycler (7900HT fast real time PCR system) (Applied Biosystems), performing an initial AmpErase Uracil N-glycosylase (UNG) activation for two minutes at 50°C, dsDNA denaturation activation for 10 min at 95°C, 40 cycles of 15 s at 95°C and 1 min at 60°C. Results were analyzed using the 2.3 SDS Software (Applied Biosystems) and RQ Manager 1.2 (Applied Biosystem). The mRNA levels (2−ΔCt) of each gene were determined calculated by subtracting the Ct value for *PPIA*, *GADPH*, *RLPO* from the Ct value for *TNF-α*, *IL-6*, *IL-8*, *IL-1ß* or *PTGS2*.

### Cell viability MTT Assay

VERO cells were seeded at 2.5x10^4^ cells/ml in 96 well-plates and treated with Mn-L1 or Mn-L2 at different concentrations (1, 3, 10, 30 and 100 μM) for 48 hours as previously described [16].

### Determination of TNF-α and IL-6 cytokines

The protein concentrations of IL-6 and TNF-α in human THP-1 macrophage culture supernatants or in mouse serum were determined by using double-antibody sandwich ELISA for human or mouse proteins (R&D Systems, Minneapolis), according to the manufacturer’s instructions.

### Western blotting

Cell lysates were prepared as described before [16–17]. Briefly, macrophages were washed twice and scraped in PBS, centrifuged, resuspended in lysis buffer (25 mM TRIS, 2 mM EDTA, 2% SDS), and sonicated. The total lysate protein concentration was determined using the Pierce BCA protein assay reagent according manufacturer’s instructions.

The lysates in sample loading buffer (20μg of protein) were resolved by SDS-PAGE and transferred onto nitrocellulose membranes. After the membranes were reversibly stained with 0.1% Ponceau Red solution, they were blocked with 3% nonfat dry milk dissolved in TBST solution (10 mmol/L Tris-HCl, pH 7.5, 100 mmol/L NaCl, and 0.1% Tween 20), incubated with the different antibodies and visualized with enhanced West Pico or West Femto chemiluminescence detection system (ECL, Pierce) according to the manufacturer’s instructions

### LPS-Induced endotoxemia in mice

Male OF1 mice weighing 30 g (Charles River, Criffa S.A., Barcelona, Spain) were bled and maintained under a continuous 12 hour light—12 hour dark cycle in specific pathogen-free conditions. The mice were fed a standard laboratory diet and water ad libitum. Animals were monitored twice a day and, in order to minimize suffering, were sacrificed by carbon dioxide inhalation followed by cervical dislocation when moribund or physical signs of pain were observed. All animal care procedures were supervised and approved by the Universitat Rovira i Virgili Animal Welfare Committee. All animal procedures conformed to EU Directive 86/609/EEC and recommendation 2007/526/EC regarding the protection of animals used for experimental and other scientific purposes, enacted under Spanish law 1201/2005.

The lethal endotoxemic test was performed as reported previously [18, 19]. Mice were randomly divided in standard cages (5 animals per cage) into three groups (n = 10 mice/group). The number of mice was selected in order to assess statistical significance and experimental reproducibility. All mice were injected intraperitoneally (ip) with LPS (30 mg/Kg), a dose that has demonstrated to be effective in causing death due septic shock in mice [20]. Mn-L1, Mn-L2 in saline solution or saline solution alone (control group) were intravenously (iv) administered for 4 days to mice at 1 mg/kg/day starting 24 hours before the LPS injection. Survival was monitored for 7 days, with survivors sacrificed at the end of the experiment by carbon dioxide inhalation.

To determine IL-6 and TNF-α serum levels mice were intraperitoneally (ip) challenged with LPS 10 mg/Kg as previously described [21]. Briefly, mice (n = 5 mice/group) were injected intravenously (iv) with Mn-L1 and Mn-L2 in saline solution at 1 mg/kg/ or with saline solution alone (control group) 24 hours and just before the LPS (10 mg/Kg) ip injection. Four hours after the LPS injection, blood samples were obtained by cardiac puncture from each mouse previously anaesthetised by inhalation with sevoflurane.

For the histology and immunohistochemistry analysis mice (n = 5 mice/group) were treated as above with 5 mg/Kg of LPS. The livers were collected 24 hours after the LPS injection.

### Histological examination and immunohistochemistry

Liver tissue was fixed immediately in 4% buffered formalin, embedded in paraffin, and cut into 4 μm sections. Histological examination was performed in H&E-stained sections. For immunohistochemistry, liver macrophages were detected using rat anti-mouse F4/80 antibody (1:1000) following previously described procedures (22). Goat anti-rat (1:200) (ref. Ba-9400, Vector, Peterborough, UK) was used as secondary antibody and the signal was amplified using the Vectastin ABC kit (Vector, Peterborough, UK) and detected with DAB+ (Dako, Barcelona, Spain). Macrophage-positive area was quantified using AnaliSYS software (Soft Imaging System, Munster, Germany).

### Statistical analysis

The data shown are a summary of the results from at least three experiments and are represented as the mean ± S.E.M. Statistical evaluation of the results was performed by one-way ANOVA with Bonferroni adjustment as part of the software package SPSS version 15.0 (LEAD technologies, USA) and plotted using GraphPad Prism version 5.0b (GraphPad Software Inc.,USA). The results were considered significant at a value of p<0.05.

## Results

### Mn-L1 and Mn-L2 anti-inflammatory activity in human macrophages

**Fig 2:**
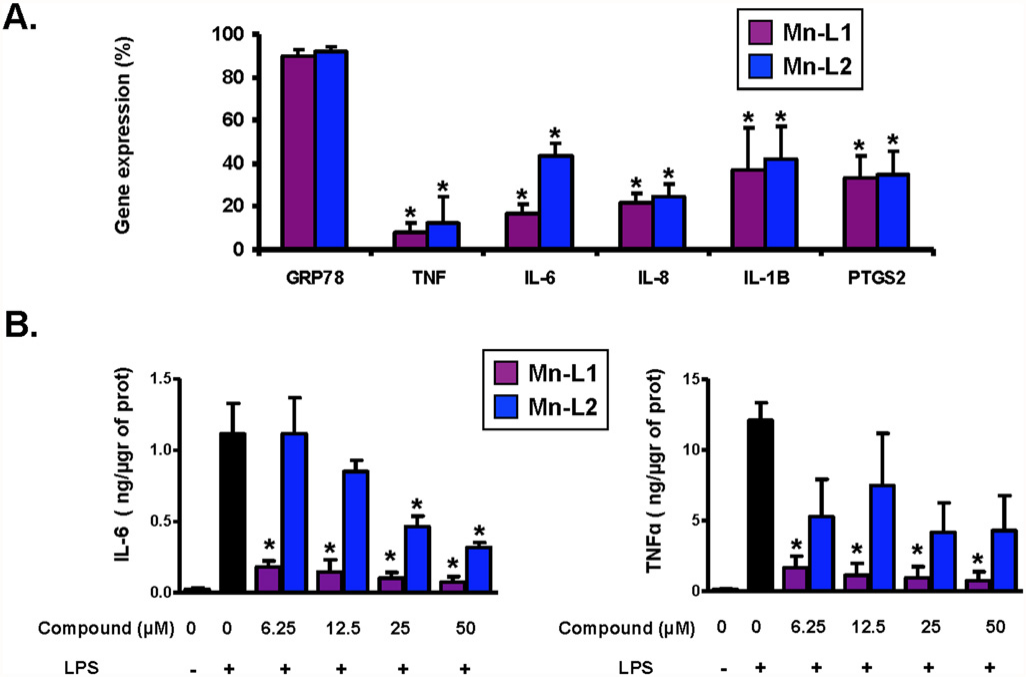
Mn-L1 and Mn-L2-inhibited LPS-induced pro-inflammatory gene expression in THP1 human macrophages. **(A) Inhibition of pro-inflammatory gene expression in macrophages by Mn-L1 and Mn-L2**. THP-1 macrophages were treated for 3 hours with 25 μmol/L of Mn-L1 or Mn-L2, the medium was removed and the cells were incubated one hour with fresh medium containing 100 ng/ml LPS. The mRNA levels were determined by quantitative real time PCR. The values are represented as percentages of the only LPS-stimulated control group. LPS 100% values (mean ± SE) were 5.5 ± 0.9; 63.58 ± 3.7; 74.12 ± 4.4; 13.83 ± 7.7.42 ± 5; and 13.15 ± 4 for GRP78, TNF-α, IL-6, Il-1ß, IL-8 and PTGS2 respectively. **(B) Inhibition of the production of pro-inflamatory TNF-α and IL-6 in macrophages by Mn-L1 and Mn-L2**. THP-1 macrophages were treated for 3 hours as explained in the figure. After 16 hours’ incubation with 500 ng/ml LPS, TNF-α, and IL-6 protein expression was determined in the cell culture medium. The data of three separate experiments performed in duplicate are shown. * P < 0.01 compared with LPS-treated group.

Frequently, molecules with antioxidant properties or free-radical scavengers at the same time present anti-inflammatory activity, suggesting that the highly antioxidant SOD mimetics Mn-L1 and Mn-L2 may also have additional anti-inflammatory properties [2, 10, 11]. We first checked the anti-inflammatory activity of Mn-L1 and Mn-L2 in macrophage cells, which are key cellular components of the inflammatory process, for which purpose we used THP-1 human macrophages which are widely employed in anti-inflammatory drug-screening studies [23, 24]. We first assessed if Mn-L1 and Mn-L2 could hamper the known induction of pro-inflammatory genes such as *TNF-α*, *IL-6*, *IL-8*, *IL-1ß*, and *PTGS2* by LPS. We observed that both compounds Mn-L1 and Mn-L2 at 25 μM significantly attenuated the LPS-induced mRNA expression of all the pro-inflammatory genes tested: *TNF-α*, *IL-6*, *IL-8*, *IL-1ß*, and *PTGS2* (Fig. 2A). For the LPS gene induction we used 100 ng/mL of LPS and one hour’ incubation which were optimal (S1 Fig.)

Second, we have measured the protein concentration of TNF-α and IL-6 in the THP-1 cell culture medium to determine if the down regulation of pro-inflammatory genes at mRNA level was also taking place at protein level. In this case Mn-L1 significantly lowered both IL-6 and TNF-α protein concentrations relative to the LPS control but, Mn-L2 only reduced IL-6 significantly at doses higher than 25 μM and was unable to significantly lower TNF-α concentration (Fig. 2B).

In addition, we have checked the toxicity of Mn-L1 and Mn-L2 in cultured cells by an MTT cell viability assay using Vero kidney epithelial cells, which are widely used for this purpose [25]. Both Mn-L1 and Mn-L2 did not affect the viability of these cells when tested at concentrations as high as 100 μM (data not shown).

### Mn-L1 anti-inflammatory activity in mice

**Fig 3:**
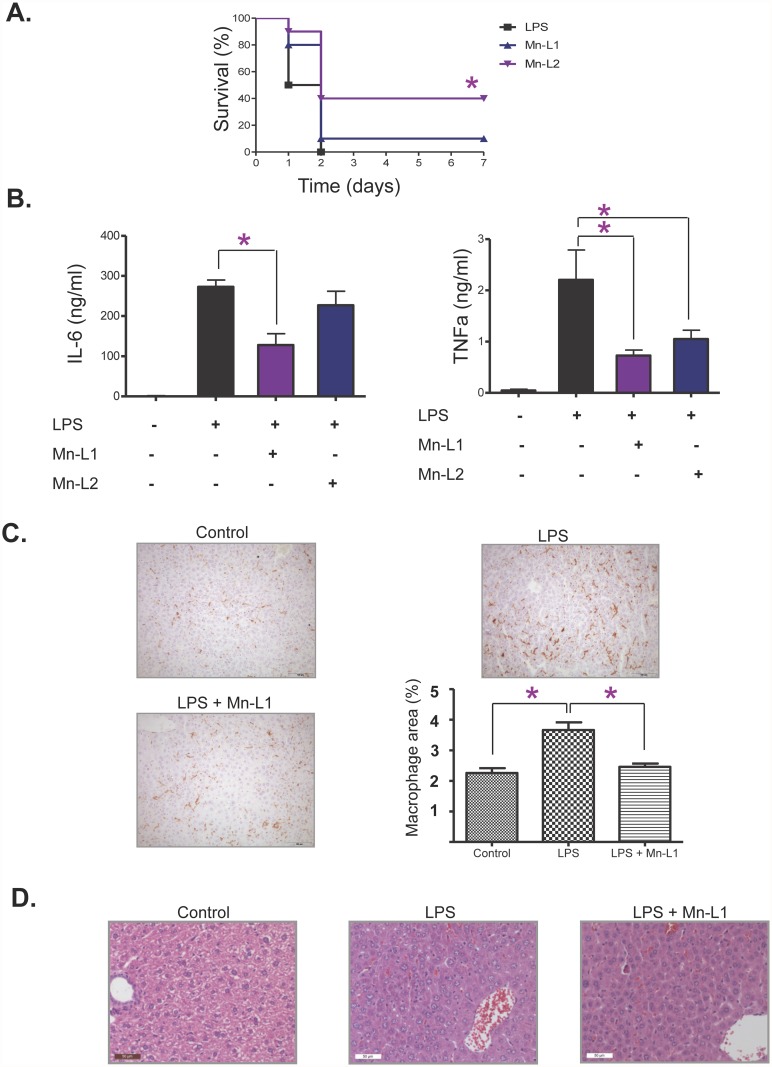
Mn-L1 and Mn-L2 anti-inflammatory activity in whole mice. **(A) Mn-L1 protected mice from a lethal endotoxemic dose of LPS**. Survival data (%) were analyzed by using the Kaplan-Meier method and log rank test. * P < 0.05 versus the LPS-treated group. **(B) Inhibition of LPS induction of TNF-α and IL-6 concentration in serum**. The graphs show the serum levels of TNF-α (right panel) and IL-6 (left panel) after the different treatments with LPS, Mn-L1 or Mn-L2 as explained under the ordinate axis. Data are presented as mean ± SEM. **(C) Mn-L1 attenuates Kupffer cell LPS activation**. The panels show the immunohistochemistry for F4/80 and the graph the quantification of positive macrophage area. *P values < 0.05. Data are represented as mean ± SEM. **(D) Mn-L1 effect in LPS liver injury**. Liver injury was determined by histological examination on H&E-stained sections. Control untreated mice; LPS (mice treated with LPS alone) and LPS + Mn-L1 (mice treated with LPS and Mn-L1).

We have examined the anti-inflammatory potential of Mn-L1 and Mn-L2 in an LPS mice model of sepsis (the systemic inflammatory response to severe microbial infection), which is extensively used as an experimental animal model replicating this condition [26]. When we challenged all the mice with a lethal dose of LPS (30 mg/Kg), we observed that all mice (10/10) in the control group died in 48 hours, 4/10 survived in the group treated with Mn-L1 (1 mg/kg) (P = 0.0128), and 1/10 in the Mn-L2- (1 mg/kg) treated group (Fig. 3A).

Since pro-inflammatory cytokines play a pivotal role in the pathogenesis of sepsis [27, 28], we next measured the concentrations of IL-6 and TNF-α in serum from mice challenged with LPS (10 mg/Kg), we observed that while both compounds at 1mg/kg significantly reduced the serum levels of TNF-α, only Mn-L1 was able to significantly reduce the serum levels of IL-6 (Fig. 3B).

Furthermore, Mn-L1 significantly reduced the activation of the liver macrophages (Kupffer cells) induced by 5 mg/Kg LPS as determined by the lower tissue intensity of F4/80, a marker of macrophage activation; [22] (Fig. 3C) and Mn-L1 also seemed to ameliorate the mild histopathological liver inflammatory response produced which consisted of a slight alveolar congestion, an increase of Kupffer cells around the hepatic sinusoids, and dilated hepatic sinusoids containing red blood cells (Fig. 3D).

Interestingly, the toxicity of Mn-L1 was also low in mice, as an intravenous 5 mg/kg, dose, which is a five times higher dose than the one used in the above experiments (1 mg/kg), administered five times with a three day interval between doses, produced no deaths, clinical signs, changes in food consumption or in body and liver weights, observable gross lesions or histopathological alterations in liver (data not shown).

### Mn-L1 hampers the LPS activation of the MAP kinase signaling pathway in macrophages

**Fig 4:**
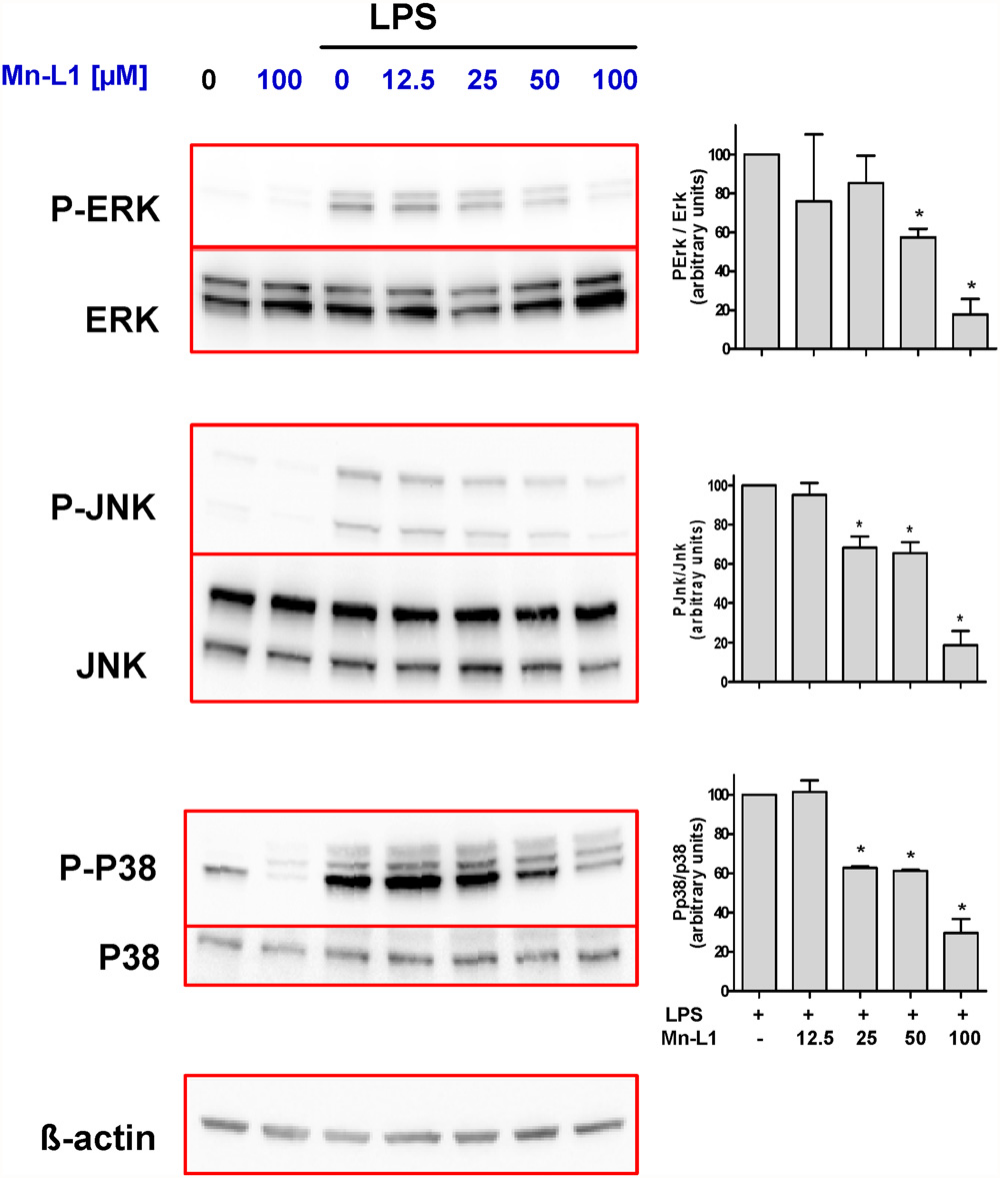
Mn-L1 inhibits LPS cellular signaling in macrophages through the MAP kinase pathway. Effects of Mn-L1 on the LPS-induced phosphorilation of MAPK kinases. THP-1 macrophages were incubated with Mn-L1 for 3 hours at the concetrations indicated in the figure and then challenged with 500 ng/ml of LPS for 1 hour. The phosphorylated and total ERK, JNK and p38 proteins were determined by Western blotting. ß-actin was used as loading control. Representative blots. (A) Representative blots and (B) densitometric evaluation (n = 3) *P < 0.01 compared to LPS-treated group.

The stimulation of macrophages by LPS leads to the activation of the MAP kinase and PI3K-Akt signaling pathways implicated in the modulation of inflammatory cytokine expression [29–31]. When we assessed the activation of these pathways, which occurs by phosphorylation at specific amino acid residues, we observed that Mn-L1 significantly decreased the phosphorylation elicited by LPS in the P38, ERK and JNK kinases of the MAP kinase pathway but did not affect the activation of the Akt pathway (Fig. 4 and S2 Fig.).

## Discussion

In the present study we have examined the anti-inflammatory properties of two Mn polyamine complexes, Mn-L1 and Mn-L2, which have high SOD mimetic activity and are able to compensate for the absence of SOD enzymes in bacteria and yeast [10, 11]. We report here that the SOD mimetic complex Mn-L1, which showed consistent anti-inflammatory activity in the different assays performed, significantly protected mice from an LPS-induced endotoxemia, which suggests that Mn-L1 complex could be of therapeutic interest.

Mn-L1 provided protection against the pro-inflammatory effects induced by LPS in cultured human macrophages as well as in mice. First, Mn-L1 was able to diminish the gene expression and protein secretion of cytokines induced by LPS in THP-1 macrophages, probably due in part to the inactivation of the MAP kinase signaling pathway, a well-known pathway implicated with the Akt pathway in the modulation of pro-inflammatory cytokines in macrophages [29–31]. On the other hand, in a mouse model of sepsis, Mn-L1 significantly improved the survival of mice treated with a lethal dose of LPS. We show that Mn-L1-treated mice had lower serum levels of the pro-inflammatory cytokines TNF-α and IL-6 relative to the control mice injected with LPS alone. In addition Mn-L1 reduced the activation of the mice liver macrophages induced by LPS. The low toxicity displayed by Mn-L1 in both human cell cultures and mice using a tolerable dose for mice five times higher (5 mg/kg) than the one used in the study assays (1 mg/kg) is noteworthy.

Why did only Mn-L1, but not Mn-L2, protect mice from a lethal endotoxemic challenge? Although Pytren4Q (L1) and Pytren2Q (L2) are isomers, they produce two Mn^II^ complexes with different coordination environments; while one of them (Mn-L1) completes its hexacoordination with a water molecule, the other (Mn-L2) involves the quinoline nitrogen in the coordination with the metal. Interestingly these features lead to different SOD activity. While the *k*_cat_ for Mn-L2 is 3.0 x 10^6^ M^-1^ s^-1^ the *k*_cat_ for Mn-L1 is five times higher (1.5 x 10^7^ M^-1^ s^-1^), only one order of magnitude below the purified SOD enzyme (1.0 x10 ^8^) and similar to the one reported for the relevant well studied, pentaazamacrocycle M40403 (2.0 x 10^7^ M^-1^ s^-1^). The higher Mn-L1 SOD activity may be related to the presence of an H_2_O molecule in its active site, a characteristic that is also present in the native enzyme [10, 11]. Although both Mn-L1 and Mn-L2 supported the growth of SOD-deficient yeast and bacteria to a similar extent, contrary to Mn-L1, Mn-L2 did not protect fish embryos from oxidative stress [11], or, in stimulated macrophages, reduce the secretion of TNF-α, and only reduced IL-6 at the highest doses tested. Interestingly Mn-L2 has showed lower activity both *in vitro* and *in vivo* in most of the assays performed. Furthermore, in the endotoxemic mice model contrarily to L1-Mn, Mn-L2 was not able to reduce the serum level of IL-6. Interestingly, although mice models of septicemia only partially reproduce the human disease [32], a high level of IL-6 in the serum of patients predicts a poor outcome in human septicemia [33–36].

In summary, there is growing evidence that Mn^II^ metal complexes of nitrogenated organic compounds have therapeutic potential in human pathologies involving superoxide production [2,6]. Interestingly, we found that Pytren4Q-Mn (Mn-L1) contrary to its isomer Pytren2Q-Mn (Mn-L2) showed consistent anti-inflammatory activity in cellular and animal settings protecting human macrophages and mice from the inflammatory effects produced by LPS. Although more in-depth studies are needed focused in the more complex human conditions, Mn-L1’s effective anti-inflammatory activity, low toxicity, water solubility, and ease of production suggest that it is worth taking into consideration for future pharmacological studies, although to date candidate agents intended to block the inflammatory response tested in mouse models of sepsis have failed in critically ill patients [32].

## Supporting Information

S1 Fig Time-course and dose-response of LPS in THP1-differentiated macrophages. Macrophages stimulation was checked at three different times (0.5, 1, and 3 hours using three doses of LPS (50, 100 and 1000 ng/ml).
(PPT)

S2 Fig Mn-L1 did not affect cellular signaling in macrophages through the Akt kinase pathway. Lack of effect of Mn-L1 on the LPS-induced phosphorilation of Akt and Ikkα/ß kinases in THP-1 macrophages. THP-1 macrophages were incubated with Mn-L1 for 3 hours at the concetrations indicated in the figure and then challenged with 500 ng/ml of LPS for 1 hour. The protein levels of phosphorylated and total Akt and Ikkα/ß were determined by Western blotting. ß-actin was used as loading control. Representative blots. (A) Representative blots and (B) densitometric evaluation (n = 3)
(PPT)
