# Supplementary figures and images for: Significant In Vivo Anti-Inflammatory Activity of Pytren4Q-Mn a Superoxide Dismutase 2 (SOD2) Mimetic Scorpiand-Like Mn (II) Complex
Source: PLoS One. 2015 Mar 5;10(3):e0119102. doi: 10.1371/journal.pone.0119102 (PMC4351122; doi:10.1371/journal.pone.0119102)

## Slide 1
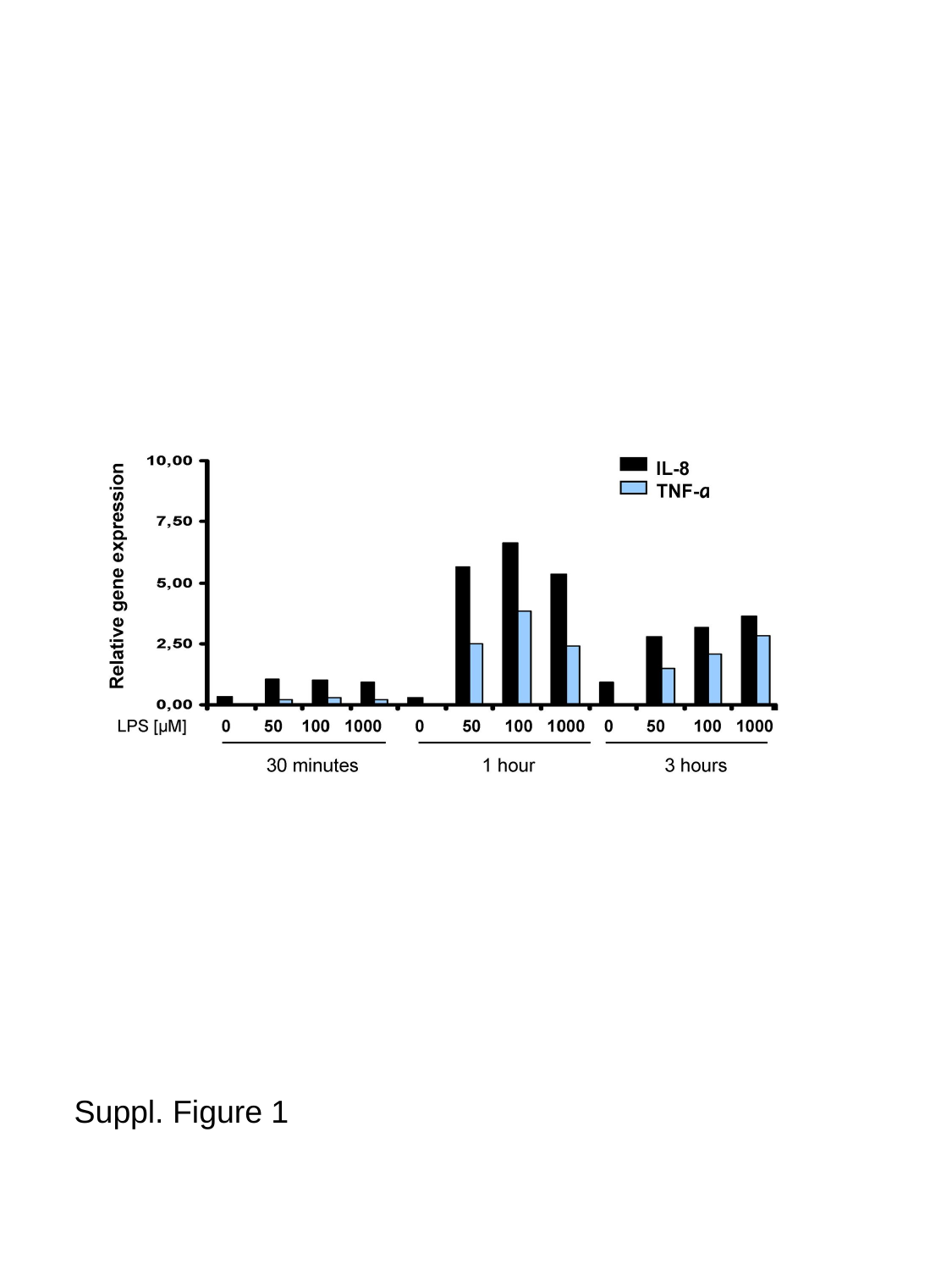

Suppl. Figure 1

Supplement: S1 Fig — Macrophages stimulation was checked at three different times (0.5, 1, and 3 hours using three doses of LPS (50, 100 and 1000 ng/ml). (PPT) [file pone.0119102.s001.ppt]

## Slide 1
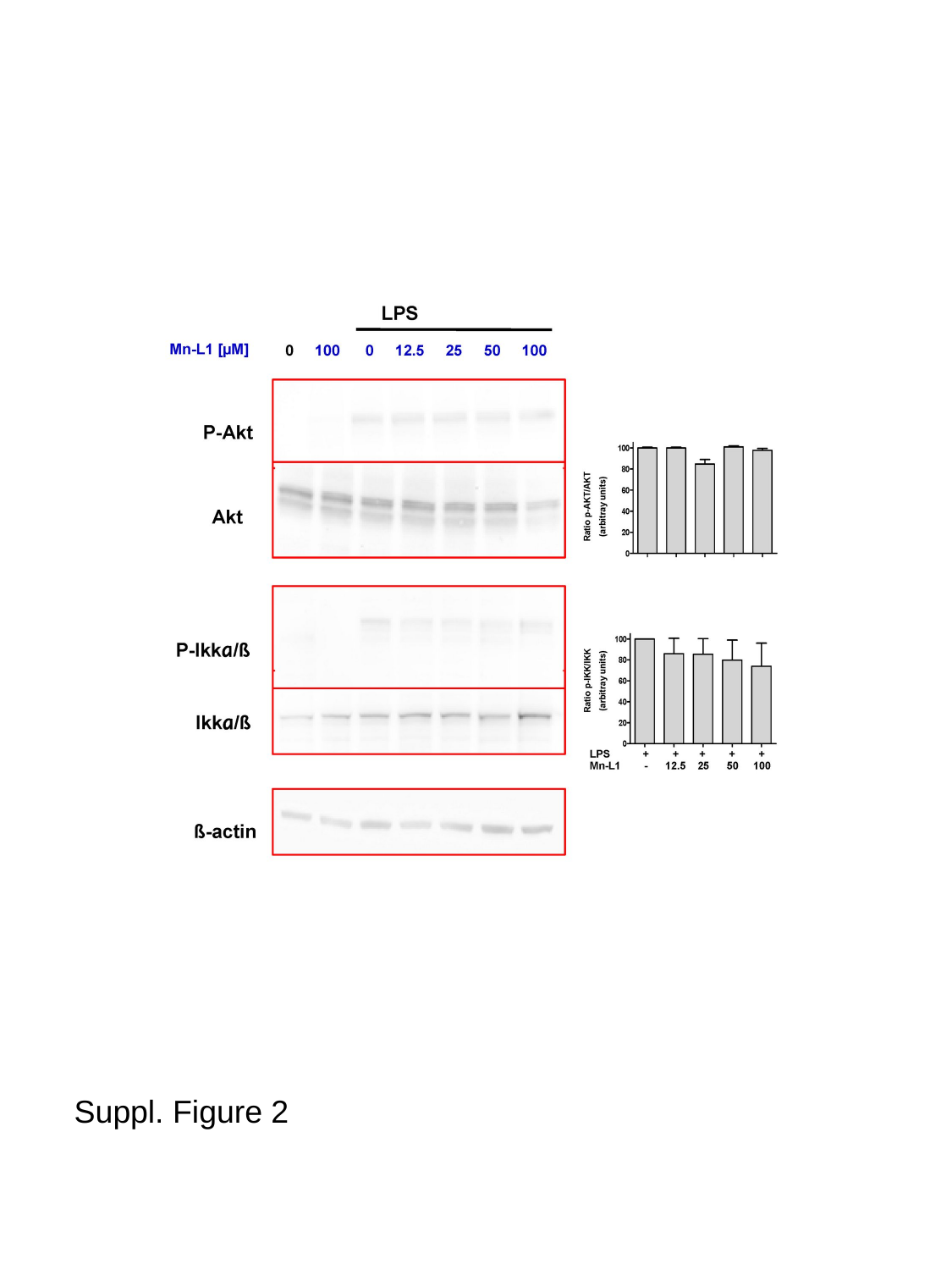

Suppl. Figure 2

Supplement: S2 Fig — Lack of effect of Mn-L1 on the LPS-induced phosphorilation of Akt and Ikkα/ß kinases in THP-1 macrophages. THP-1 macrophages were incubated with Mn-L1 for 3 hours at the concetrations indicated in the figure and then challenged with 500 ng/ml of LPS for 1 hour. The protein levels of phosphorylated and total Akt and Ikkα/ß were determined by Western blotting. ß-actin was used as loading control. Representative blots. (A) Representative blots and (B) densitometric evaluation (n = 3) (PPT) [file pone.0119102.s002.ppt]
